# Analysis of mortality in a pooled cohort of Canadian and German uranium processing workers with no mining experience

**DOI:** 10.1007/s00420-017-1260-9

**Published:** 2017-09-22

**Authors:** Lydia B. Zablotska, Nora Fenske, Maria Schnelzer, Sergey Zhivin, Dominique Laurier, Michaela Kreuzer

**Affiliations:** 1 Department of Epidemiology and Biostatistics, University of California, San Francisco, San Francisco, CA USA; 2 Department of Radiation Protection and Health, Federal Office for Radiation Protection, Neuherberg, Germany; 3 French National Institute of Health and Medical Research, INSERM, Paris, France; 4 Institute for Radiological Protection and Nuclear Safety, IRSN, Fontenay-Aux-Roses, France

**Keywords:** Uranium, Radon, Gamma radiation, Cohort study, Risk assessment

## Abstract

**Purpose:**

Long-term health risks of occupational exposures to uranium processing were examined to better understand potential differences with uranium underground miners and nuclear reactor workers.

**Methods:**

A cohort study of mortality of workers from Port Hope, Canada (1950–1999) and Wismut, Germany (1946–2008) employed in uranium milling, refining, and processing was conducted. Poisson regression was used to evaluate the association between cumulative exposures to radon decay products (RDP) and gamma-rays and causes of death potentially related to uranium processing.

**Results:**

The pooled cohort included 7431 workers (270,201 person-years of follow-up). Mean RDP exposures were lower than in miners while gamma-ray doses were higher than in reactor workers. Both exposures were highly correlated (weighted rho = 0.81). Radiation risks of lung cancer and cardiovascular diseases (CVD) in males were increased but not statistically significant and compatible with risks estimated for miners and reactor workers, respectively. Higher RDP-associated CVD risks were observed for exposures 5–14 years prior to diagnosis compared to later exposures and among those employed <5 years. Radiation risks of solid cancers excluding lung cancer were increased, but not statistically significant, both for males and females, while all other causes of death were not associated with exposures.

**Conclusions:**

In the largest study of uranium processing workers to systematically examine radiation risks of multiple outcomes from RDP exposures and gamma-rays, estimated radiation risks were compatible with risks reported for uranium miners and nuclear reactor workers. Continued follow-up and pooling with other cohorts of uranium processing workers are necessary for future comparisons with other workers of the nuclear fuel cycle.

**Electronic supplementary material:**

The online version of this article (doi:10.1007/s00420-017-1260-9) contains supplementary material, which is available to authorized users.

## Introduction

Nuclear fuel cycle workers are exposed to a variety of hazardous materials (ATSDR 2013; IARC 2012). The main steps of the cycle involve uranium exploration and mining, followed by uranium milling, processing and refining in preparation for uranium conversion, enrichment and fuel manufacturing, and completed by exploitation of nuclear reactors and nuclear fuel reprocessing.

Uranium underground miners are primarily exposed to internal ionizing radiation from radon decay products (RDP) via inhalation. To a lesser extent, they are also exposed to uranium ore dust and to external gamma-rays, both of which are important to consider for risk to organs other than lungs. Workers involved in uranium milling, refining and processing (defined thereafter as “uranium processing workers”) account for ~10–15% of ~500,000 workers employed worldwide in the nuclear fuel cycle in the last 40–50 years (UNSCEAR 2010). The process starts with milling the ore by crushing and grinding to liberate minerals, then leaching it with sulfuric acid to dissolve the uranium oxides. The solution is then processed to recover the uranium and to form a uranium oxide concentrate called ‘yellowcake’ containing more than 80% uranium (Bigu and DuPort 1992). To increase the proportion of uranium-235 which is capable of undergoing fission and producing energy in a nuclear reactor to between 3.5 and 5%, the yellowcake concentrate undergoes the process of isotope separation and refinement to concentrate (enrich) uranium-235 isotope relative to other uranium isotopes. As a result, uranium workers are exposed to dust, acids, lime, solvents, noise and heat. In addition, uranium minerals are always associated with more radioactive elements such as radium and radon in the ore which arise from the radioactive decay over millions of years. Radium decays by emitting high-LET alpha-radiation, which has been found to be carcinogenic to humans (IARC 2001). Uranium processing workers also come in contact with other types of radiation (e.g., gamma-ray, long-lived radionuclides from uranium ore dust) and non-radioactive (e.g., fine or silica dust) exposures from the ore dust, but less to RDP exposures, typical for uranium underground miners. Average annual effective radiation doses in this group have been reported at 10 millisievert (mSv) compared to <5 mSv for other workers of the fuel cycle (Bouville and Kryuchkov 2014). Several studies reported substantially higher cumulative lifetime occupational gamma-ray exposures for uranium processing workers (Kreuzer et al. 2015; Zablotska et al. 2013) compared to external radiation exposures of nuclear reactor workers (Cardis et al. 2007; Muirhead et al. 2009). At the same time, cumulative RDP exposures have been reported (Kreuzer et al. 2015; Zablotska et al. 2013) as several times lower than internal exposures of uranium underground miners (NRC 1999). Thus, there is an emerging consensus that exposures of workers in the uranium processing industry are substantially different from those of uranium underground miners, enrichment workers or nuclear reactor workers, and that processing workers should be carefully evaluated in separate studies.

To date, epidemiological studies of uranium underground miners (NRC 1999) and uranium enrichment workers (Chan et al. 2010; Guseva Canu et al. 2011; McGeoghegan and Binks 2000; Yiin et al. 2017; Zhivin et al. 2016) have reported increased risks of lung cancer. Large pooled studies of nuclear reactor workers showed significantly increased risks of solid cancers and leukemia, (Cardis et al. 2007; Gillies and Haylock 2014; Muirhead et al. 2009; Richardson et al. 2015; Schubauer-Berigan et al. 2015) and, more recently and controversially, of cardiovascular (CVD) (Azizova et al. 2015; Muirhead et al. 2009) and non-malignant respiratory diseases (Azizova et al. 2017; Muirhead et al. 2009). Only a few studies have examined risks of exposures in the uranium processing industry (Boice et al. 2008; Dupree-Ellis et al. 2000; Dupree et al. 1987; Guseva Canu et al. 2010; Kreuzer et al. 2015; Nusinovici et al. 2010; Pinkerton et al. 2004; Richardson et al. 2013; Silver et al. 2013; Zablotska et al. 2013) and reported contradictory results, necessitating further research in this area. In comparison to the general population, uranium processing workers had higher mortality rates from lung cancer (Pinkerton et al. 2004; Silver et al. 2013; Zablotska et al. 2013), lymphatic and hematopoietic, particularly non-Hodgkin lymphoma (NHL) and multiple myeloma (MM), cancers (Guseva Canu et al. 2010; Kreuzer et al. 2015; Pinkerton et al. 2004; Richardson et al. 2013; Silver et al. 2013), and kidney or bladder cancers (Boice et al. 2008; Dupree-Ellis et al. 2000; Kreuzer et al. 2015; Richardson et al. 2013; Zablotska et al. 2013). Recent studies have reported increased risks of CVD (Dupree et al. 1987; Guseva Canu et al. 2012; Kreuzer et al. 2015; Nusinovici et al. 2010; Zablotska et al. 2013) and non-malignant respiratory diseases (Boice et al. 2008; Dupree et al. 1987; Pinkerton et al. 2004), but overall mortality was similar to the general population.

Few studies conducted dose–response analyses of uranium processing workers with individual radiation doses (Dupree-Ellis et al. 2000; Kreuzer et al. 2015; Silver et al. 2013; Zablotska et al. 2013). Uranium processing workers from the Port Hope radium and uranium refinery and processing plant in Canada and from the Wismut facilities in Germany were exposed to similar radiation and non-radiation factors. These two studies are the only ones to allow estimation of risks among uranium processing workers not only from RDP exposures but also from gamma-rays. Recent risk analyses of these cohorts were based on similar exposure estimation methods (Kreuzer et al. 2015; Zablotska et al. 2013). In a study of 3000 uranium millers and processors from Port Hope, a small but not statistically significant increase in risk of lung cancer associated with RDP exposures was reported (Zablotska et al. 2013). A statistically significant increase in mortality from all cancers associated with RDP exposures, primarily due to lung cancer, was found in a study of 4054 Wismut workers (Kreuzer et al. 2015). This paper presents the results of the pooled analysis of the data from the Port Hope and Wismut studies. Outcomes of interest were determined by potential uranium-target organs among uranium processing workers, including lung and bronchi, liver, kidney, bone, upper respiratory tract, and lymphatic and hematopoietic tissues. Possible associations with CVD outcomes from low-dose RDP and gamma-ray exposures were also investigated. Additional exploratory analyses were conducted to estimate radiation risks among females involved in uranium milling and processing (355 and 270 workers, Port Hope and Wismut, respectively).

## Materials and methods

### Port Hope

#### Cohort characteristics and follow-up

Port Hope cohort’s materials and methods have been described previously (Zablotska et al. 2013) and are briefly summarized below. Information on 3338 potential study subjects came from the personnel records of the Cameco Corporation Port Hope Conversion Facility (Port Hope). For inclusion in the study, workers had to be employed at Port Hope during the ages of 15–75 years sometime between 1932 and 1980, had their last contact after 1940, and had to be alive at start of mortality follow-up in 1950. All workers were included regardless of duration of employment. We used National Dose Registry information and Eldorado’s personnel records to exclude Port Hope workers with any mining experience, leaving a cohort for analysis of 3000 workers.

The nominal roll file was linked to the Canadian Mortality Data Base (CMDB) to ascertain mortality from 1950 to 1999. Data in the CMDB are obtained through the vital statistics system for national reporting of vital statistics data, which is considered virtually complete with under-coverage at <1% (Goldberg et al. 1993). The “alive” follow-up (1984–2000) was completed via deterministic linkage with the Historic Tax Summary file using the social insurance number (SIN). Workers, who could not be linked to the Historic Tax Summary file or the CMDB, were considered lost to follow-up and had their termination date at work recorded as the last date alive.

#### Assessment of exposures

The individual annual exposures in working-level-months (WLM) were calculated from working level (WL)^1^ estimates for each type of workplace, the proportion of employees in each occupation, and the proportion of time spent in each type of workplace by employees in each occupation. The WL estimates were based on quantities of radium present in the plant in ore and at various stages of refinement, measured radon emanation rates from various radium-bearing materials, building air volumes and estimates of air exchange rates. We did not estimate separate radium doses but used this information in the calculation of the RDP estimates.

Gamma-ray radiation was the primary type of radiation exposure at Port Hope. Film badges were used on some individuals in the late 1940s, and were worn by most radium workers and a sampling of others from mid-1947 to early 1953. Full individual external dosimetry (100% coverage) was in place by about 1970. In this analysis, personal gamma-ray doses were calculated from the average dose-rates and time on the job and expressed in mSv for each individual who had not been wearing a badge. All gamma-ray doses were whole-body effective doses. Workers who had worked in radium operations at any time were classified as radium workers, while all other workers who had never worked in radium operations were classified as uranium workers. No other individual exposures have been estimated for this cohort.

### Wismut millers

#### Cohort characteristics and follow-up

The German male Wismut uranium miners cohort study has been described previously (Kreuzer et al. 2010). It is a stratified random sample of 58,982 male former employees of the uranium mining company Wismut in East Germany, who had worked for at least 6 months during the operation period from 1946 to 1990. A similar female cohort exists including 3996 former Wismut employees. The data of both cohorts pertain to a third mortality follow-up from January 1, 1946, through December 31, 2008, with information on the vital status from local registries. Information on the underlying cause of death is based on death certificates from the Public Health offices and their archives and the autopsy files from the Wismut pathology archive. The total cohort includes workers from different types of work places (underground mines, open pit mines, surface and milling). All workers based in milling facilities, who had never worked either underground or in open pit mines, were selected, resulting in 4161 male and 270 female workers. A previous analysis of the male cohort of uranium millers excluded 107 persons with missing silica dust information (Kreuzer et al. 2015). These persons were included in the present analysis.

#### Assessment of exposures

Information on date of start of employment, date of end of employment and, for each year, type of work place, facility and job type were collected from the pay rolls for each cohort member. Exposure to radon progeny, long-lived radionuclides and external gamma radiation was determined based on a comprehensive job-exposure matrix that assigns an average annual exposure value to each facility, work place and job type. In milling facilities, first measurements of radon and external gamma radiation started in 1955, while systematic measurements in the mine-shafts were conducted since 1963 (Kreuzer et al. 2015).

### Statistical analyses

Each individual contributed person-years at risk from the later of the date of hire or the start date of follow-up to the exit date or the date of death, or the last date known alive. Start date was defined as January 1st, 1950, for Port Hope workers and January 1st, 1946, for Wismut workers. Exit date was defined as December 31st, 1999, for Port Hope workers and December 31st, 2008, for Wismut workers. The last date known alive was defined as date of last employment or contact, whichever occurred earlier.

Main analyses were based upon internal comparisons and used grouped Poisson regression analyses (Breslow and Day 1987) to estimate risks from a simple linear excess relative risk (ERR) model:

1 $${\text{Rate}}_{D} = {\text{Rate}}_{0}^{*} \left( { 1 + \left( {\beta^{*} D} \right){ \exp }\left( {\varSigma_{i} \gamma_{i} {\text{Z}}_{i} } \right)} \right).$$

where Rate_*D*_ is the rate at dose *D*, Rate_0_ is the background rate (stratified to adjust for potential confounders), *D* represents factors such as cumulative lagged continuous RDP exposure or gamma-ray whole-body dose, *Z*_*i*_ are potential risk modifying factors and *β* and *γ*_*i*_ are fit parameters. The *β* is referred to as the ERR per unit of exposure; by adding 1.0 to the ERR one obtains the relative risk per 100 WLM for RDP exposure or per one Sv for gamma-ray dose. In exploratory analyses, both gamma-ray and RDP exposure terms were included in the model simultaneously.

To examine the shape of the dose–response, a series of categorical analyses were done with cutpoints of RDP exposures and gamma-ray doses chosen to evenly distribute deaths between categories. All relative risks (RR) were calculated relative to a referent category of <0.3 WLM for RDP exposures and <0.3 mSv for gamma-ray doses.

Confounders were retained in the model if they produced a sizable (≥10%) change in the point estimate of the ERR. Potential confounders of the background rate included age at risk, calendar year, duration of employment, and predominant exposures to radium/uranium (Port Hope) and cumulative exposures to long-lived radionuclides, silica or fine dust and arsenic (Wismut cohort). The person-years at risk were cross-classified by age at risk (15–19, 20–24… 85–100 years old), calendar year at risk (in 5-year categories), total duration of employment (<6 and 6 months+),^2^ and cumulative exposure, separately for RDP exposures and gamma-ray doses. The person-year weighted mean cumulative exposure in each cross-classified cell was used in the regression analysis. RDP exposures and gamma-ray doses were lagged by 5 years to account for latency period between exposure and death. In exploratory analyses, 10-, 15- and 20-year lags were used for analyses of CVD outcomes for comparability with previous studies (Little et al. 2012).

In the original Port Hope cohort, the underlying causes of death were recoded from the original International Classification of Disease (ICD) code in use at the time of death or diagnosis to ICD-9 (World Health Organization (WHO) 1998). Deaths in the Wismut cohort have been recoded to ICD-10. ICD codes for main outcomes of interest are presented in Supplementary Table S1.

Modifying effects of several factors were examined in a model with time-window analyses which allows to evaluate the effect of exposures accrued in one time period while adjusting for the effect of exposures accrued at other time periods (Richardson and Ashmore 2005):

2 $${\text{Rate}}_{D} = {\text{Rate}}_{0}^{*} (1 + \beta^{*} (r_{5 - 14} + \theta_{15 - 24} r_{15 - 24} + \theta_{25 + } r_{25 + } ){ \exp } (\varphi_{\text{age at risk}} + \gamma_{\text{exposure rate}} )).$$

where 5-year cumulative lagged RDP or gamma-ray exposure (*r*) is partitioned into time windows (exposures 5–14, 15–24, and 25+ years previously), and *φ* and *γ* represent estimates of modifications to the dose–response by categories of age at risk and exposure rate, respectively. Exposure rate was estimated as a time-dependent ratio of cumulative dose and cumulative duration of exposure (employment). In addition, based on recently published analyses of radiation-related risks of CVD, age at first exposure and duration of exposure (employment) were examined as potential modifiers of the dose–response (Zablotska et al. 2014).

Regression parameters, confidence intervals around point estimates and *P* values were estimated using the method of maximum likelihood (McCullagh and Nelder 1989) in the AMFIT module of the EPICURE software (Preston et al. 1993). Deviances of the models estimated by this method were used to assess model fits and models with smaller deviances were considered to have a better fit. Tests of statistical significance were based on the likelihood ratio test comparing the deviances of two nested models with and without exposure variables, which has a large-sample Chi-square distribution with degrees of freedom equal to the difference in the number of parameters estimated. All *P* values quoted were two-sided. Because of the form of Eq. 1, the possible values of *β* are limited by the requirement that the corresponding relative risk should not be negative. If the likelihood being sought for a point or bound estimate did not converge, the minimum value for *β* was given by −1/*D*_max_, where *D*_max_ was the maximum dose.

## Results

### Demographic and exposure characteristics

Table 1 presents the basic characteristics of the pooled cohort of uranium processing workers from the Port Hope and Wismut studies. The mean sex-specific values of lifetime RDP exposures and gamma-ray doses are presented for the cohort as a whole (*n* = 7431), and separately for females (*n* = 625) and males (*n* = 6806). RDP exposures and gamma-ray doses were not normally distributed in the two cohorts and in the pooled cohort (*P* values of all Kolmogorov–Smirnov tests <0.05). Cumulative 5-year lagged RDP exposures and gamma-ray doses were strongly correlated (person-year weighted Spearman’s rho correlation coefficient 0.97, 0.82, and 0.82, Wismut, Port Hope and pooled cohort, respectively). Male workers had significantly higher RDP and gamma-ray doses compared to female workers involved in uranium refining and processing (both *P* values from the Wilcoxon Rank Sum Test <0.001). The majority of workers were male (91.6% of the cohort). Historically, females tended to work at office jobs or as laboratory technicians.

**Table 1:** Basic characteristics of the Port Hope and Wismut cohorts

Characteristic	Port Hope (*n* = 3000)		Wismut (*n* = 4431)		Total
	Male	Female	Male	Female	
*N* (%)	2645 (88.2%)	355 (11.8%)	4161 (93.9%)	270 (6.1%)	7431 (100%)
Person-years	82,753 (87.2%)	12,103 (12.8%)	163,832 (93.4%)	11,513 (6.6%)	270,201
Lifetime^a^ RDP exposure, WLM					
Mean (median)	13.3 (0.41)	4.9 (0)	8.5 (5.2)	7.4 (4.5)	10.0 (3.0)
Range (SD)	0–627.6 (45.9)	0–62.7 (9.6)	0.01–126.9 (9.7)	0.02–44.1 (8.1%)	0–627.6 (28.6)
Lifetime^a^ gamma-ray dose, mSv					
Mean (median)	116.3 (21.1)	36.2 (2.6)	30.8 (12.3%)	31.1 (10.7)	61.5 (13.8)
Range (SD)	0–5098.8 (312.1)	0–464.7 (69.7)	0.03–667.4 (64.4)	0.04–464.7 (69.7)	0–5098.8 (197.4)

*mSv* millisieverts, *RDP* radon decay products, *WLM* working level months

^a^ Individual exposures cumulated up to the end of follow-up

There were 270,201 person-years of mortality follow-up in the pooled cohort. Average duration of follow-up was 31 years in the Wismut and 23 years in the Port Hope cohort. Average age at start of employment was 29 years (SD = 10) in Wismut workers and 30 years (SD = 11) in Port Hope workers. Workers were employed for an average of 15 years (range 0–44) in the Wismut and 6 years (range 0–46) in the Port Hope facilities. All Wismut workers were exposed to non-zero doses of RDP exposures and gamma-ray doses, while among Port Hope workers only 56.2% of workers (*n* = 1687) had any recorded RDP exposures and 94.3% (*n* = 2830 workers) had non-zero gamma-ray doses.

### Males

The person-year weighted 5-year lagged mean cumulative RDP exposure among males in the pooled cohort was 16.6 WLM (SD = 49.8), higher among Port Hope workers compared to Wismut workers (21.1 and 10.0 WLM, respectively). The person-year weighted 5-year-lagged mean cumulative gamma-ray dose was 136.8 mSv (SD = 324.5), higher among male Port Hope workers compared to Wismut workers (189.4 and 58.6 mSv, respectively). Formal tests of heterogeneity of radiation risks of various cancer and non-cancer outcomes between the cohorts indicated no statistically significant differences (all *P* > 0.05, not shown), so all new analyses were conducted in the pooled cohort.

#### Solid cancers

Radiation risks of solid cancers were increased but not statistically significant, both in analyses of RDP exposures and gamma-ray doses (Table 2). When deaths from lung cancer were excluded from the analysis, radiation risks estimates decreased for both exposures but more so for RDP exposures. Radiation risks of lung cancer mortality tended to increase with both increasing RDP exposures and gamma-ray doses, but both estimates were not statistically significant (*P* = 0.16 and *P* = 0.39, RDP exposures and gamma-ray doses, respectively, Table 2). The model for lung cancer with RDP exposures alone had a lower deviance compared to the model with gamma-ray doses only (991.2 vs. 992.4). Furthermore, addition of a second independent linear term for gamma-ray doses to the model with a linear term for RDP exposures did not significantly improve the fit of the model (*P* = 0.68).

**Table 2:** Excess risk estimates and 95% confidence intervals for RDP exposures and gamma-ray doses for selected cancer and non-cancer causes of death, combined Port Hope and Wismut cohorts, men only

Cause of death	Port Hope	Wismut	RDP exposure			Gamma-ray dose		
	1950–1999	1946–2008	ERR/100 WLM^a^	95% CI	*P* value^b^	ERR/Sv^c^	95% CI	*P* value^b^
All causes of death	2641	4161						
Solid cancer	225	408	0.23	−0.11; 0.87	0.27	0.28	<−0.25; 1.11	0.32
Solid cancer excluding lung cancer	126	245	0.09	<−0.19; 0.76	0.63	0.20	<−0.46; 1.26	0.56
Lung cancer	99	163	0.68	<−0.23; 2.45	0.16	0.43	<−0.46; 2.13	0.39
Larynx cancer	5	8	nc			nc		
Liver and biliary	4	12	nc			nc		
Kidney cancer	7	12	3.48	<−4.82; 33.7	0.41	−0.19	<0.20; 28.6	0.88
Bladder cancer	10	22	−0.16	<−0.16; 2.16	0.58	nc		
Hematological cancers^d^	24	17	−0.16	<−1.23; 2.17	0.55	nc		
All CVD	514	749	0.12	−0.05; 0.36	0.20	0.13	−0.11; 0.48	0.32
Hypertensive disease	13	36	0.13	<−0.81; 3.38	0.82	0.58	<−1.10; 5.12	0.49
IHD	346	360	0.17	−0.09; 0.34	0.18	0.21	<−0.13; 0.71	0.26
Stroke	71	181	−0.07	<−0.40; 0.52	0.72	−0.19	<−1.12; 0.50	0.39
COPD	29	59	−0.16	<−0.16; 1.41	0.60	−0.19	<−3.84; 1.40	0.59

*CI* confidence interval, *COPD* chronic obstructive pulmonary disease, *CVD* cardiovascular diseases, *ERR/Sv* excess relative risk per 1 Sv, *ERR/100 WLM* excess relative risk per 100 WLM, *IHD* ischemic heart disease, *nc* no convergence, *RDP* radon decay products

^a^ Model adjusted for calendar time, age at risk, cohort and duration of employment (<6 vs. 6+ months; Port Hope cohort only) by stratification. Gamma-ray doses were not included in the model

^b^ *P* values from the likelihood ratio test comparing nested model with and without the exposure term

^c^ Model adjusted for calendar time, age at risk, cohort and duration of employment (<6 vs. 6+ months; Port Hope cohort only) by stratification. RDP exposures were not included in the model

^d^ Includes Non-Hodgkin lymphoma, Hodgkin’s disease, multiple myeloma and leukemia

Analysis of other cancer outcomes, which could be potentially associated with uranium processing work, did not yield any significant results. In general, models with RDP exposures had smaller deviances compared to the models with gamma-ray doses, indicating a better model fit. In models with two terms for RDP exposures and gamma-ray doses, risks were due to RDP exposures only, and the fit of the model did not significantly improve with addition of the gamma-ray dose term (all *P* > 0.40, not shown).

#### Hematological cancers

The radiation risk estimates for RDP exposures and gamma-ray doses for hematological outcomes (non-Hodgkin lymphoma, Hodgkin’s disease, multiple myeloma and leukemia) were on the lower bound of the −1/*D*_max_, which produced negative estimates. RDP and gamma-associated risks of leukemia were null (not shown).

#### Non-cancer outcomes

The estimates of radiation risks of mortality due to all CVD causes were similar for RDP exposures and gamma-ray doses (Table 2, *P* = 0.20 and *P* = 0.32, RDP exposures and gamma-ray doses, respectively). In models with two terms for RDP exposures and gamma-ray doses, risks were primarily due to RDP exposures (ERR/100WLM = 0.24 and ERR/Sv = −0.19 from the combined model with two linear terms, not shown). The fit of the model with RDP exposures did not improve with addition of the gamma-ray dose either as a linear term (*P* = 0.58) or as log-linear term (*P* = 0.13). In general, model deviances were comparable for RDP exposures and gamma-ray doses (Table 3), which is to be expected due to a high correlation between these exposures. The lowest deviances were estimated for models with unlagged and 5-year lagged exposures, although differences between models with 5 and 20-year lags were very small (Table 3). Radiation risks for IHD were somewhat higher compared to the risks estimated for all CVD, although still not statistically significant (Table 2).

**Table 3:** Deviances of various risks models and lag times for CVD mortality

Exposure	Lag time, years	Deviance	ERR/100 WLM^a^
RDP exposure	0	2737.128	0.12
	5	2737.205	0.12
	10	2737.425	0.11
	15	2737.542	0.11
	20	2737.355	0.13
			ERR/Sv^b^
Gamma-ray doses	0	2737.889	0.13
	5	2737.890	0.13
	10	2738.079	0.12
	15	2738.172	0.12
	20	2738.163	0.13

*CVD* cardiovascular diseases, *deviance* −2 log likelihood of the fitted model from the maximum likelihood estimation procedure, *ERR/Sv* excess relative risk per 1 Sv

^a^ Model adjusted for calendar time, age at risk, cohort and duration of employment (<6 vs. 6 + months; Port Hope cohort only) by stratification. Gamma-ray doses were not included in the model

^b^ Model adjusted for calendar time, age at risk, cohort and duration of employment (<6 vs. 6+ months; Port Hope cohort only) by stratification. RDP exposures were not included in the model

**Fig. 1:**
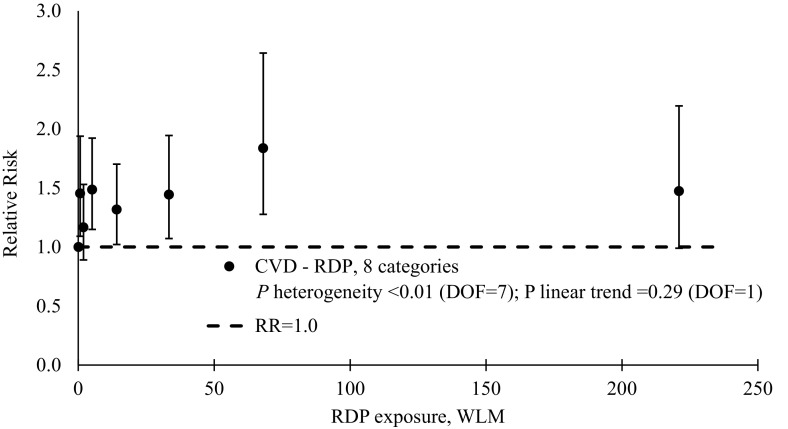
Plot of relative risks of CVD mortality by mean RDP exposure, pooled Port Hope and Wismut cohort. The referent relative risk is 1.0

**Fig. 2:**
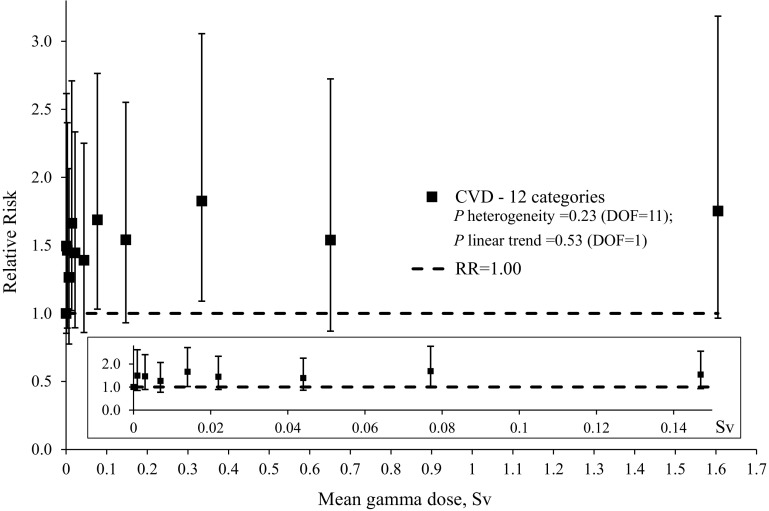
Plot of relative risks of CVD mortality by mean gamma-ray dose, pooled Port Hope and Wismut cohort. The referent relative risk is 1.0. The inset shows detail for dose range 0–0.15 Sv

Several exploratory categorical analyses were conducted to further examine the positive, although not statistically significant, finding for CVD mortality. Significant heterogeneity was observed between category-specific RRs for CVD mortality in models with RDP exposures (*P* < 0.01, Table 4), but the test for linear trend was not statistically significant (*P* = 0.29). In contrast, both tests for heterogeneity of category-specific RRs for CVD mortality in models with gamma-ray doses (*P* = 0.23, Table 5) and the test for linear trend were not statistically significant (*P* = 0.53). In general, relative risks for both exposures were increased by 20–75% compared to the reference categories (<0.3 WLM or <0.3 mSv). Figures 1 and 2 show plots of RDP- and gamma-ray-associated risks and suggests a pattern of increased risks, irrespective of exposure and categorization methods.

**Table 4:** Relative risk estimates and 95% confidence intervals for CVD mortality by category of cumulative RDP exposure, male Port Hope and Wismut workers

Dose categories, WLM	Mean dose, WLM	Deaths		Person-years		RR^a, b^	95% CI
		#	%	#	%		
0–0.34	0.1	97	8	74,669	30	1	
0.35–1.09	1	106	8	26,251	11	1.45	1.09; 1.94
1.10–2	2	154	12	38,564	16	1.17	0.89; 1.53
3–7	5	272	22	47,801	19	1.49	1.15; 1.92
8–23	14	408	32	44,660	18	1.32	1.02; 1.70
24–49	33	120	10	9270	4	1.44	1.07; 1.94
50–99	68	59	5	2865	1	1.84	1.28; 2.64
100–623	221	47	4	2506	1	1.47	0.99; 2.20
Total	17	1263	100	246,586	100		

*CVD* cardiovascular diseases, *CI* confidence interval, *DOF* degrees of freedom, *RR* relative risk, *WLM* working level months

^a^ *P* heterogeneity ≤0.01 (DOF = 7); *P* linear trend 0.29 (DOF = 1)

^b^ Model adjusted for calendar time, age at risk, cohort and duration of employment (<6 vs. 6+ months; Port Hope cohort only) by stratification. Gamma-ray doses were not included in the model

**Table 5:** Relative risk estimates and 95% confidence intervals for CVD mortality by category of cumulative gamma-ray doses, male Port Hope and Wismut workers

Dose categories, Sv	Mean dose, Sv	Deaths		Person-years		RR^a, b^	95% CI
		#	%	#	%		
0–0.00034	0.0001	20	2	42,649	17	1	
0.00035–0.0014	0.001	37	3	17,549	7	1.50	0.85–2.62
0.0015–0.005	0.003	113	9	34,293	14	1.46	0.89–2.40
0.005–0.010	0.007	129	10	35,729	14	1.27	0.78–2.06
0.010–0.018	0.014	148	12	27,812	11	1.66	1.02–2.71
0.018–0.032	0.022	212	17	32,155	13	1.45	0.89–2.33
0.032–0.058	0.044	206	16	22,188	9	1.39	0.86–2.25
0.058–0.102	0.077	130	10	12,946	5	1.69	1.03–2.76
0.102–0.240	0.147	106	8	11,081	4	1.54	0.93–2.55
0.240–0.500	0.334	85	7	5849	2	1.83	1.09–3.06
0.500–1.000	0.651	42	3	2692	1	1.54	0.87–2.72
1.000–5.097	1.605	35	3	1643	1	1.75	0.97–3.19
Total	0.137	1263	100	246,586	100		

*CVD* cardiovascular diseases, *CI* confidence interval, *DOF* degrees of freedom, *RR* relative risk, *Sv* sievert

^a^ *P* heterogeneity = 0.23 (DOF = 11); *P* linear trend = 0.53 (DOF = 1)

^b^ Model adjusted for calendar time, age at risk, cohort and duration of employment (<6 vs. 6+ months; Port Hope cohort only) by stratification. RDP exposures were not included in the model

Although the splitting of the cumulative RDP exposure into three time windows since exposure did not significantly improve the model fit (Table 6, *P* = 0.62), we observed a monotonic decrease in risk with increasing time since exposure. CVD mortality risks were not modified by exposure rate, age at risk or age at first RDP exposure, but a significant heterogeneity in radiation risks for duration of employment (*P* = 0.01) was estimated, with those employed 0–4 years having two times higher risks compared to those employed 5 or more years (RR = 2.07, 95% CI: 1.03, 4.14, not shown).

**Table 6:** Interaction models for CVD mortality by cumulative RDP exposure, male Port Hope and Wismut workers

Parameter	Number of deaths	Parameter estimate and 95% CI^a^	*P* value^b^	Deviance
Continuous doses lagged by 5 years	1263	0.12 (−0.05 to 0.35)^c^	0.19	3521.315
Continuous doses lagged by 20 years	1263	0.14 (−0.05 to 0.40)^c^	0.16	3521.128
Time-window analysis	1263			
WLM 5–14 previously		1	0.62	3533.458
WLM 15–24 previously		0.23 (<−0.28 to 0.99)^d^		
WLM 25+ previously		0.09 (−0.18 to 0.56)^d^		
Interaction terms for time-window analyses				
Exposure rate, WLM/year (continuous)		0.40 (0.09–1.81)^e^	0.94	
Age at risk, years (continuous)		0.96 (0.91–1.01)^e^	0.99	
Age at first RDP exposure, years (continuous)		1.00 (1.00–1.00)^e^	0.96	
Duration of employment, years (continuous)		0.76 (0.59–0.98)^e^	0.01	

*CI* confidence interval, *CVD* cardiovascular diseases, *ERR/100 WLM* excess relative risk per 100 WLM, *nc* no convergence, *RDP* radon decay products

^a^ Model adjusted for calendar time, age at risk, cohort and duration of employment (<6 vs. 6+ months; Port Hope cohort only) by stratification. Gamma-ray doses were not included in the model

^b^ *P* values from the test of heterogeneity of category-specific relative risks

^c^ ERR/100 WLM

^d^ RR for time since exposure window compared to reference (exposures 5–14 years previously)

^e^ ERR/100 WLM for time since exposure window 5–14 years and effect modifying variable

### Females

Person-time weighted 5-year lagged cumulative RDP exposures were lower among female workers compared to male workers (6.5 and 16.6 WLM, respectively). Gamma-ray doses were almost fourfold lower among females compared to males (40.8 and 136.8 mSv, respectively). While RDP exposures for female workers were similar in Port Hope and Wismut cohorts (6.7 and 6.3 WLM, respectively), gamma-ray doses were twofold higher for female Port Hope workers (51.4 and 30.9 mSv, respectively). The radiation risks of solid cancer were increased both for RDP exposures and for gamma-ray doses, although not statistically significant (Table 7). Increased risks were primarily due to increased risks of breast and colon cancer, and when these were excluded from analysis, risks of solid cancer became negative, both for RDP exposures and gamma-rays. While an estimate of radiation risk was negative for all CVD mortality, it was increased for IHD, although not statistically significant.

**Table 7:** Excess risk estimates and 95% confidence intervals for RDP exposures and gamma-ray doses for selected cancer and non-cancer causes of death, combined Port Hope and Wismut cohorts, women only

Cause of death^d^	Port Hope	Wismut	RDP exposure			Gamma-ray dose		
	1950–1999	1952–2008	ERR/100 WLM^a^	95% CI	*P* value^b^	ERR/Sv^c^	95% CI	*P* value^b^
All causes of death	270	354						
Solid cancer	24	24	1.96	<−1.95, 10.7	0.36	2.58	−2.79, 15.2	0.39
Solid cancer excl lung cancer	17	19	2.60	<−2.08, 13.9	0.29	7.90	<−2.38, 33.0	0.09
Solid cancer excl colon and breast cancer	16	19	−0.43	<−3.29, 5.85	0.82	−1.10	<−4.20, 6.89	0.64
Lung cancer	7	5	−1.16	<−8.93, 30.0	0.81	nc		
Breast cancer	5	2	5.37	<−10.3, 281	0.53	17.30	<9.8, 366	0.22
All CVD	36	59	−0.14	<−1.79, 2.77	0.90	−0.69	<−3.12, 3.58	0.68
IHD	22	26	1.32	<−1.94, 9.46	0.49	1.20	<−3.52, 11.9	0.68

*CI* confidence interval, *CVD* cardiovascular diseases, *ERR/Sv* excess relative risk per 1 Sv, *ERR/100 WLM* excess relative risk per 100 WLM, *IHD* ischemic heart disease, *nc* no convergence, *RDP* radon decay products

^a^ Model adjusted for calendar time, age at risk, cohort and duration of employment (<6 months vs. 6+ months; Port Hope cohort only) by stratification. Gamma-ray doses were not included in the model

^b^ *P* values from the likelihood ratio test comparing nested model with and without the exposure term

^c^ Model adjusted for calendar time, age at risk, cohort and duration of employment (<6 months vs. 6+ months; Port Hope cohort only) by stratification. RDP exposures were not included in the model

^d^ Models did not converge or had negative radiation risk estimates for all other outcomes

## Discussion

The follow-up of uranium processing workers is essential to improve understanding of radiation risks associated with employment in the nuclear processing industry and to ensure that radiation protection programs appropriately protect workers’ health. This work presents the results from one of the largest cohort analyses comprised of workers exposed to a unique combination of RDP exposures and gamma-ray doses as a result of the milling, processing and refining of uranium. RDP exposures were broadly similar in the two cohorts, but gamma-ray doses almost four-fold higher among male Port Hope workers. Overall, RDP exposures were highly correlated with gamma-ray doses. We determined that radiation risks of all cancer and non-cancer outcomes were similar in the two cohorts, indicating that the cohorts were suitable for pooling. Overall, radiation risks of lung cancer due to RDP exposures and of CVD due to both RDP exposures and gamma-ray doses among males were not significant, but similar in size to risks reported for uranium miners [National Research Council (NRC) 1999] and nuclear reactor workers (Muirhead et al. 2009; Richardson et al. 2015).

Several previous studies of nuclear reactor workers (Richardson et al. 2015; UNSCEAR 2008) reported significantly increased risks of all solid cancers and all solid cancers excluding lung cancer due to gamma-ray exposures. Several recent studies reported significantly increased risks of solid cancer in relation to RDP exposures (Kreuzer et al. 2015; Rage et al. 2015) which were primarily due to increased risk of lung cancer. In the analyses presented here, radiation risks of solid cancers for males were increased but not statistically significant both for RDP exposures and for gamma-ray doses. When lung cancer cases were excluded, risk estimates decreased, indicating that increased solid cancer risks were driven by lung cancer risks.

Studies of uranium processing workers reported increased mortality from lymphatic (Guseva Canu et al. 2010; Kreuzer et al. 2015; Pinkerton et al. 2004; Richardson et al. 2013; Silver et al. 2013), intestinal (Silver et al. 2013), pleural cancers (Guseva Canu et al. 2010) and non-malignant respiratory (Boice et al. 2008; Dupree et al. 1987; Pinkerton et al. 2004) and renal diseases (Dupree-Ellis et al. 2000; Pinkerton et al. 2004) in comparison to the general population. A significant dose-dependent increase in risks of intestinal cancer was reported for uranium processing workers from the Fernald Feed Materials Production Center in the US (Silver et al. 2013). In the current analysis, none of these cancer sites were found to be significantly related to workers’ RDP exposures or gamma-ray doses.

Dose-dependent increases in risk of CVD from gamma-ray doses have been reported in the study of uranium miners and other uranium workers from the Wismut facilities (Kreuzer et al. 2013) while risks from RDP exposures were negative. In contrast, RDP-associated risks of CVD were significantly increased in French uranium miners (Nusinovici et al. 2010) and in the Mayak cohort of workers occupationally exposed to external gamma-rays and/or internally to alpha-particles from incorporated alpha-emitting radioisotopes (Azizova et al. 2015). In the current pooled analysis, increased risks of CVD mortality were similar for RDP exposures and gamma-ray doses, with slightly lower model deviances for the former and slightly higher point estimates for the latter. Time-window analyses of RDP-associated risks of CVD with age at risk and exposure rate effect modification terms did not provide a better fit compared to a conventional model, but a monotonic decrease in risk with increasing time windows since exposure was found. Significant heterogeneity in radiation risks for duration of employment requires further exploration. Radiation risk of IHD due to RDP exposures was also increased in females, but not statistically significant.

This was the first study to evaluate radiation risks of women employed in the uranium processing industry. Increased risks of solid cancer were primarily due to increased risks of colon and breast cancer. Unusually high risk estimates for some outcomes among women could be related to the small size of the female sample and the small numbers of deaths and should be explored in larger pooled analyses.

One of the strongest advantages of this study is the long-term follow-up with essentially complete ascertainment of mortality. The large size of the cohort (*n* = 7431), percentage of workers deceased (39.5%) and the length of follow-up (50 years in the Port Hope and 63 years in the Wismut cohort) were substantially greater compared to other studies. In contrast to the majority of published studies of uranium processing workers based on analyses of mortality in comparison to the general population, detailed individual annual exposure information was available and dose–response analyses could be conducted. Comparison of risks from RDP and gamma-ray exposures provided a complementary view of the effects of uranium milling and processing occupational exposures on the risk of cancer and non-cancer outcomes.

The most important limitation of this study is the limited statistical power due to very low RDP exposures and low gamma-ray exposures. This could be addressed through further follow-up and pooling of the two cohorts with other cohorts from similar uranium processing operations (Laurent et al. 2016). No data were available on exposures to long-lived radionuclides, arsenic, fine or silica dust in the Port Hope cohort. However, recent analysis of Wismut millers indicated that any increase in mortality risks was primarily due to RDP exposures and gamma-ray radiation and not to long-lived radionuclides from uranium ore dust (Kreuzer et al. 2015). In addition, preliminary dose calculations for the Wismut millers indicated that absorbed organ doses from inhalation of alpha-emitting long-lived radionuclides from uranium ore dust were very low, on average about 3 mGy for the lung, and 1 mGy for liver and red bone marrow (Kreuzer et al. 2015). There was no information on behavioral risk factors. For smoking to confound the RDP-associated risk of lung cancer, smoking habits should be correlated with both RDP exposure and lung cancer. Mortality and incidence of tobacco-related cancers in the Port Hope cohort were similar to the general population of Canada, suggesting that smoking was not substantially elevated relative to the general population (Zablotska et al. 2013). Furthermore, a case–control study of Canadian underground uranium miners reported no association between smoking and RDP exposure (L’Abbe et al. 1991).

No assessment of RDP or gamma-ray dose measurement errors on the risk estimates was conducted in both cohorts. In the Port Hope cohort, RDP concentration estimates were based on plant inventories of radiation-bearing materials, published or otherwise known values of radon emanation rates from various materials, building volumes and estimated air exchange rates. The material inventories likely varied day-to-day but over the year would have been exact and, therefore, not a major contributor to error in annual average concentrations. Random errors in radon emanation rates and building volumes cannot be excluded but are expected to be small. The equilibrium factor relating RDP to radon concentrations is a function of the air exchange rate and could be a significant contributor to errors in RDP exposures. In the Wismut cohort, a comprehensive job-exposure-matrix (JEM) based on expert rating in the early years and on ambient measurements in the later years was used to estimate exposure. This may involve measurement error. Sources of uncertainties in exposure assessment in the Wismut cohort and their effects on the risk estimates are currently under investigation.

We had limited data on incorporation and internal exposures to radium and uranium for Port Hope workers from urinalyses tests conducted since the mid-1960s, which could not be used for internal dose calculations. We also did not have information on quartz or fine silica dust exposures for Port Hope workers, which have been shown to independently increase the risk of lung cancer. However, a small fraction of Port Hope employees before 1955 would have had some dust exposure and the quartz content of that dust would have been much less than that from some of the other uranium properties operating at the time.

There was no individual gamma-ray external dosimetry in the early years of operation in both cohorts, so all early exposures were estimated. For some early years there was missing data on inventories in specific steps of the operation, but a statistical analysis of film badge readings in the Port Hope cohort through these years showed that variance was small and this was not a significant contributor to error (Zablotska et al. 2013). Of greater importance was the variation in individual work habits and the question of whether an individual was actually present in the assumed location in the specific time period. But, since the gamma-ray dose estimates were done based on annual averages, the likely errors would be small. Measurement errors in exposure estimation almost certainly decreased with calendar time; thus recent workers should have lower mean errors than earlier workers. A fourfold difference in mean gamma-ray doses among male uranium processing workers in the two cohorts is notable and is probably due to very high early exposures in the Port Hope cohort.

## Conclusions

In this analysis of a cohort of workers exposed to uranium milling and processing with detailed annual exposure information, over 90% of workers were followed-up for at least 20 years, allowing sufficient time for occupationally-induced cancers and non-cancers to develop. Small but not statistically significant increases in risks of solid cancer, lung cancer and CVD due to RDP exposures and gamma-ray doses among males were found. Radiation risks of solid cancers, breast cancer and IHD were increased but not statistically significant among females. All other causes of death were not found to be associated with occupational RDP exposures and gamma-ray doses among males and females. RDP exposures and gamma-ray doses were highly correlated. Continued follow-up of the cohorts and pooling with other cohorts of workers exposed to byproducts of radium and uranium processing could provide valuable insights into risks from occupational uranium exposures and gamma-ray doses, and suspected differences in risk with uranium miners and nuclear reactor workers.

## Electronic supplementary material

Below is the link to the electronic supplementary material.

Supplementary material 1 (PDF 95 kb)

## Footnotes

1 The concentration of RDP per liter of air that would result in the ultimate release of 1.3 × 10^5^ MeV of potential alpha-particle energy. WLM is equivalent to one working month (170 h) in a concentration of 1 WL.

2 Total duration of employment was split at 6 months, as risk drops after 6 months but then remains constant. Similar phenomena have been previously observed in other studies (Howe et al. 1988).
