# Supplementary material for: Analysis of mortality in a pooled cohort of Canadian and German uranium processing workers with no mining experience
Source: Int Arch Occup Environ Health. 2017 Sep 22;91(1):91–103. doi: 10.1007/s00420-017-1260-9 (PMC5752750; doi:10.1007/s00420-017-1260-9)
Supplement: Supplementary file 1 — Supplementary material 1 (PDF 95 kb) [file 420_2017_1260_MOESM1_ESM.pdf]

*International Archives of Occupational and Environmental Health*

**Analysis of mortality in a pooled cohort of Canadian and German uranium processing workers with no mining experience**

by Lydia B. Zablotska[1], Nora Fenske[2], Maria Schnelzer[2], Sergey Zhivin[3], Dominique Laurier[4], Michaela Kreuzer[2]

[1]Department of Epidemiology and Biostatistics, University of California, San Francisco, San Francisco, CA, USA; [2]Federal Office for Radiation Protection, Department of Radiation Protection and Health, Neuherberg, Germany; [3]French National Institute of Health and Medical Research, INSERM, Paris, France; [4]Institute for Radiological Protection and Nuclear Safety, IRSN, Fontenay-aux-Roses, France.

*Corresponding Author* Lydia B. Zablotska, Email: Lydia.Zablotska@ucsf.edu; Phone: (415) 476-4673; ORCID: 0000-0002-0778-1108.

**Supplementary Table 1** List of International Classification of Diseases (ICD) codes for causes of death studied in the pooled analysis of Port Hope and Wismut cohorts.[a]

| | **Port Hope** | **Wismut** |
|---|---|---|
| **Causes of Death** | **ICD-9** | **ICD-10** |
| **Cancer Causes of Death** | | |
| Liver | 155 | C22.0, C22.2-C22.4, C22.7, C22.9 |
| Biliary | 155.1-156.9 | C22.1, C23-C24 |
| Larynx | 161 | C32 |
| Lung and Bronchus | 162.2-162.9 | C34 |
| Bones and Joints | 170 | C40-C41 |
| Urinary Bladder | ~~188~~ | ~~C67~~ |
| Kidney, Renal Pelvis, Ureter | 189 | C64-C66 |
| Hodgkin Lymphoma | 201 | C81 |
| Non-Hodgkin Lymphoma | 200, 202.0-202.2, 202.8-202.9 | C82-C85, C96.3 |
| Myeloma | 203.0, 238.6 | C90.0, C90.2 |
| Chronic Lymphocytic Leukemia | 204.1 | C91.1 |
| Other Leukemia | 204.0, 204.2-204.9, 205, 207-208 | C90.1, C91.0, C91.2-C91.9, C92-C95 |
| | | |
| **Non-Cancer Causes of Death** | | |
| Hypertension | 401-405 | I10-I15 |
| Ischemic Heart Disease | 410-414, 429.2 | I20-I25, I51.6 |
| Cerebrovascular Diseases | 430-438 | I60-I69 |
| Other Diseases of Heart | 390-398, 402, 404, 410-448 | I00-I09, I11, I13, I20-I51, I70-I78 |

| | | |
|---|---|---|
| Chronic Obstructive Pulmonary Disease and Allied Conditions | 466, 490-491, 496 | J40-J42, J44 |

[a]http://seer.cancer.gov/codrecode/1969+_d04162012/index.html
